# Allergic Contact Dermatitis Caused by Silver Sulfate Present in the Wound Dressing Following Total Knee Arthroplasty: An Unusual Case

**DOI:** 10.7759/cureus.84351

**Published:** 2025-05-18

**Authors:** Hatim Mohammed Alshareef, Waheeb Abed Alharbi, Naeem Khalaf N. Aljabbab, Mohammed A Alkhotani, Hassan O Bogari, Ahmed Elbarbary

**Affiliations:** 1 Orthopedic Surgery, King Fahad Armed Forces Hospital, Jeddah, SAU; 2 Orthopedic Surgery, King Abdulaziz University Hospital, Jeddah, SAU; 3 College of Medicine, King Saud Bin Abdulaziz University for Health Sciences, Jeddah, SAU

**Keywords:** allergic contact dermatitis (acd), postoperative surveillance, silver dressing, surgical wound dressing, total knee arthroplasty (tka)

## Abstract

Total knee arthroplasty (TKA) is a widely performed orthopedic surgical procedure for severe cases of osteoarthritis, providing significant pain relief and improved function. Here, we report a case of a 55-year-old female who underwent TKA on her left knee and presented later with erythema, itching, and burning sensation on the left knee, raising a suspicion of allergic contact dermatitis (ACD) following the use of a silver sulfate-containing wound dressing after TKA. The patient was managed successfully with a conservative approach, receiving medical treatment after the allergy was reported, and was discharged with only minimal neurological residuals. Although ACD to silver sulfate is uncommon, maintaining a high index of suspicion, supported by comprehensive history taking and examination, is crucial for early detection and effective management to mitigate potential complications.

## Introduction

Total knee arthroplasty (TKA), also known as total knee replacement, is a surgical intervention aimed at replacing a damaged or deteriorated knee joint with an artificial one, typically crafted from metal and plastic components [1]. Postoperative wound management is crucial in preventing complications such as infection and delayed healing. Silver-containing dressings are commonly used due to their antimicrobial properties, reducing the risk of surgical site infections. However, despite their benefits, silver-based products can occasionally lead to adverse skin reactions. Silver allergy is a hypersensitivity reaction to silver, causing symptoms such as itching, redness, swelling, or a rash on the skin when in contact with silver items. This type of allergy is typically a form of allergic contact dermatitis (ACD) [2]. Differentiating ACD from irritant contact dermatitis (ICD) presents significant diagnostic challenges due to their overlapping clinical features, such as erythema, edema, and vesiculation. ACD is an immune-mediated hypersensitivity reaction triggered by allergens, while ICD results from direct damage to the skin by irritants. The overlapping complicates the diagnosis, often relying on patient history, exposure assessment, and patch testing [3]. A rare case of allergic contact dermatitis induced by silver nitrate in a widely used special patch test marker has been reported previously [4]. However, to our knowledge, no cases have been reported on silver sulfate-containing wound dressings. ACD due to silver sulfate is an uncommon but important complication in wound care. This case report describes a patient who developed allergic contact dermatitis following the use of a silver sulfate-containing wound dressing after total knee arthroplasty.

## Case presentation

**Figure 1:**
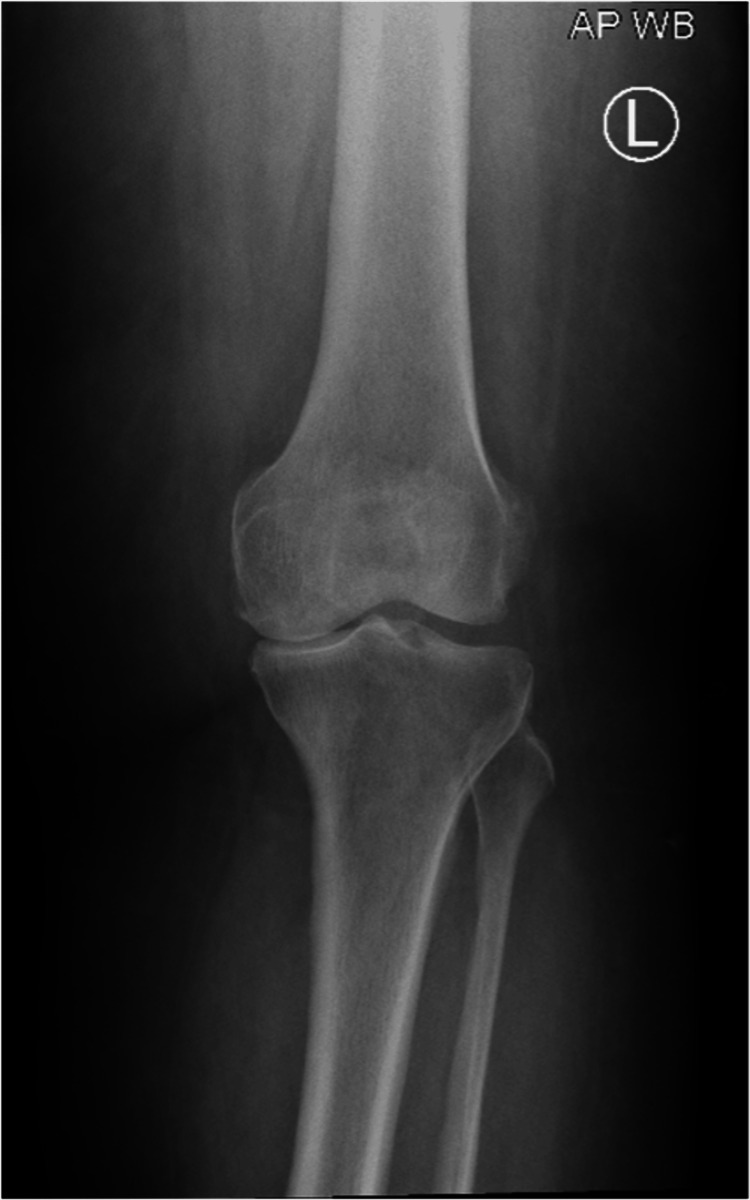
Left knee X-ray before total knee arthroplasty showing osteoarthritis

**Figure 2:**
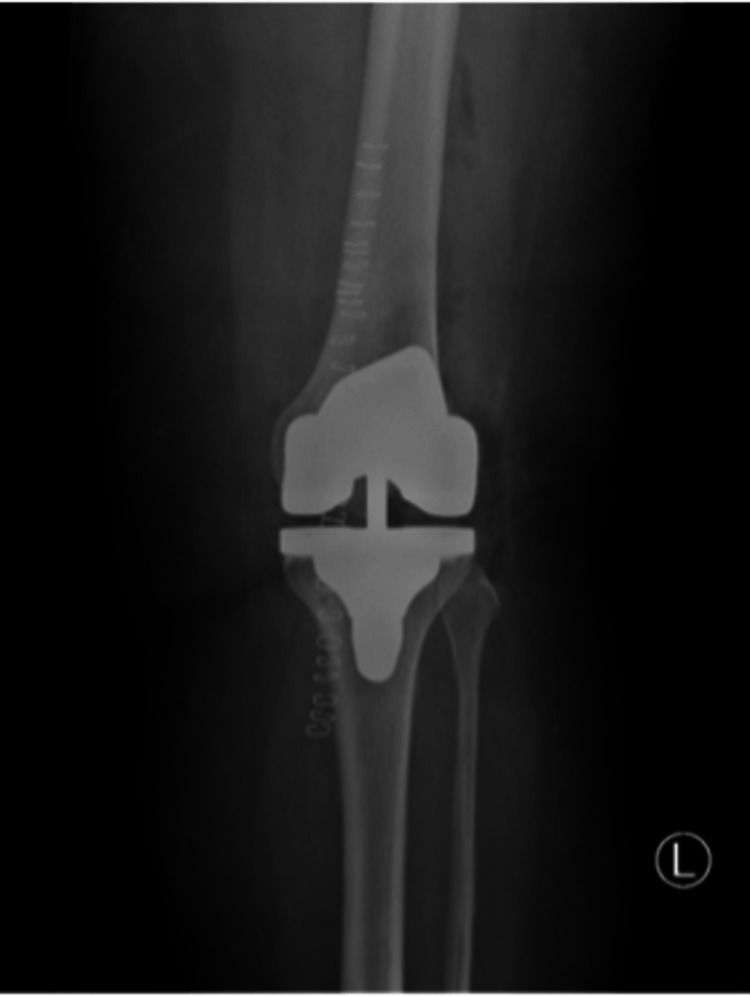
Left knee X-ray after total knee arthroplasty, with no postoperative complications

A 55-year-old woman with a history of diabetes mellitus and hypertension, without any known allergies, presented with erythema, itching, and burning sensation on the left knee. The patient had osteoarthritis and had undergone total knee arthroplasty on her left knee seven days prior to presentation (Figure 1). The patient’s scheduled surgery was postponed due to a high hemoglobin A1C (HbA1c) level and was rescheduled when the HbA1c level was reduced to below 7.5%. After the surgery, the patient applied Mepilex Border Post-Op Ag to her left knee for postoperative surgical wound care for seven consecutive days. Postoperative orthopedic findings were normal (Figure 2). Dermatological examination findings were also normal, except for erythema, edema, burning, and itching around the borders of the area on which the Mepilex Border Post-Op Ag had been applied. Mepilex Border Post-Op Ag is a wound dressing used for the management of exuding wounds such as surgical and traumatic wounds. It consists of polyurethane, silicone, polyacrylate, cotton, viscose, polyester, polyolefin, and silver sulfate that equals to 1.2 mg/cm^2^ silver.

**Figure 3:**
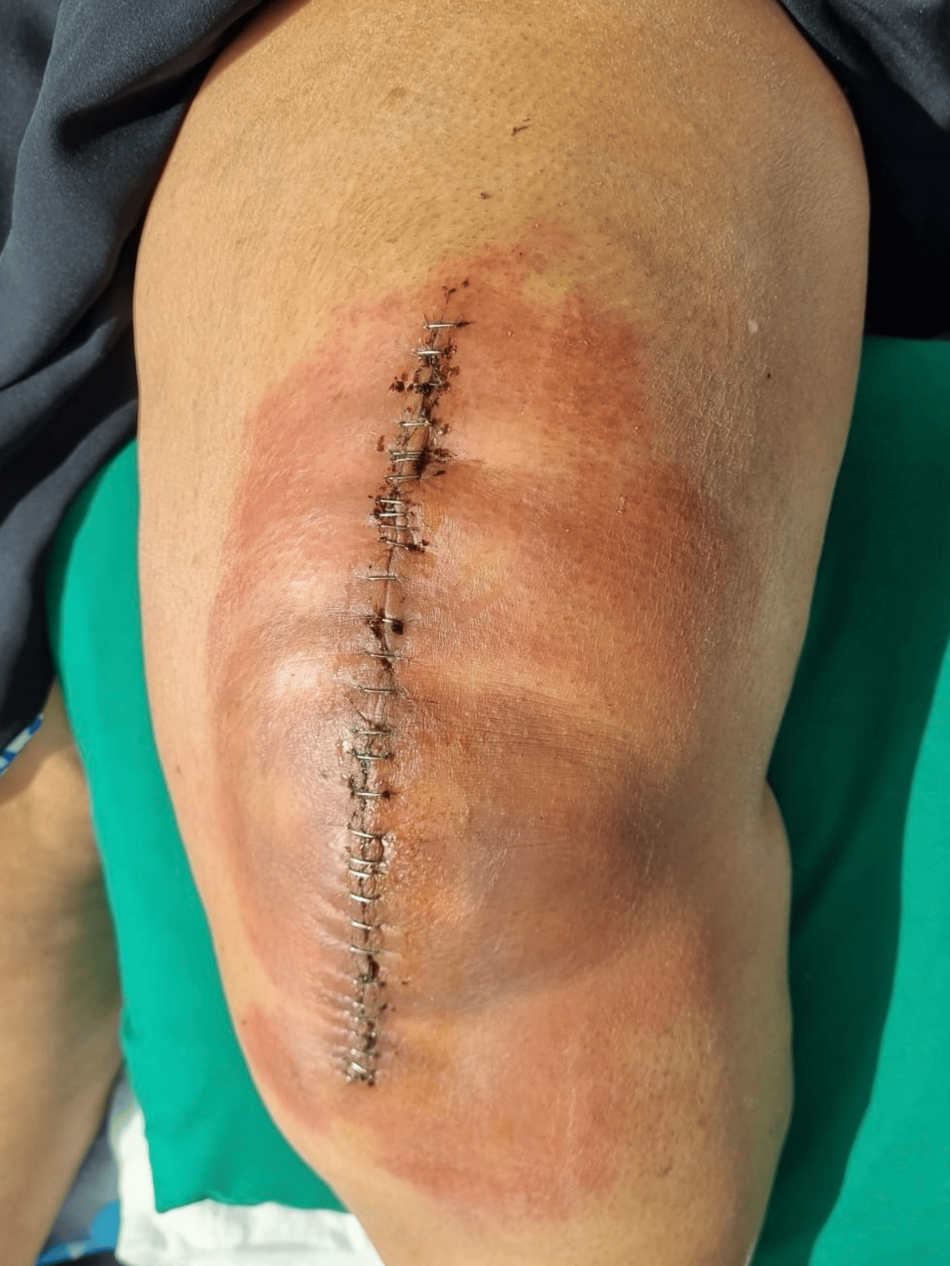
Wound at one week postoperatively, showing dermatitis

**Figure 4:**
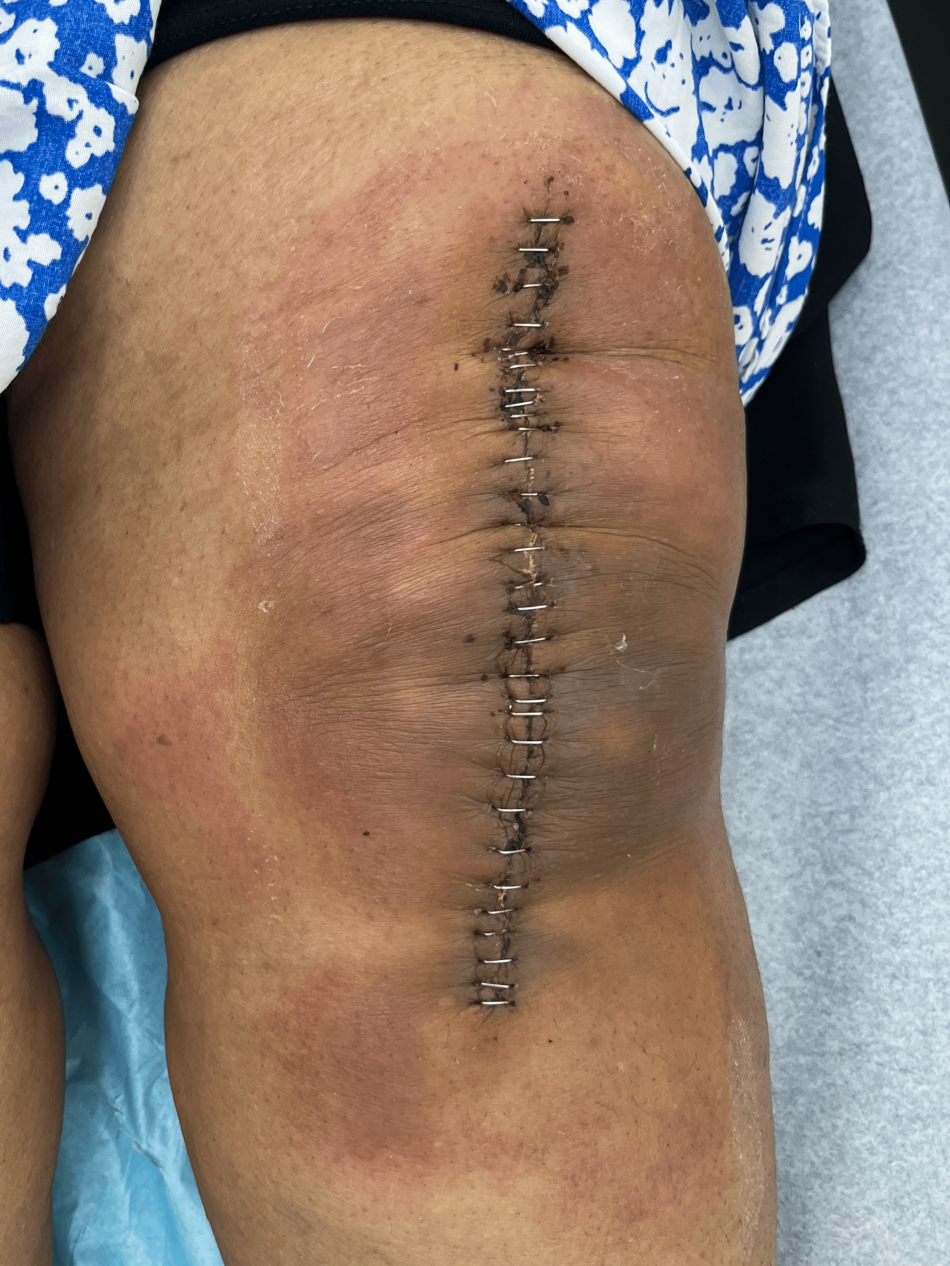
Wound at two weeks postoperatively, improving after changing the silver-containing dressing

**Figure 5:**
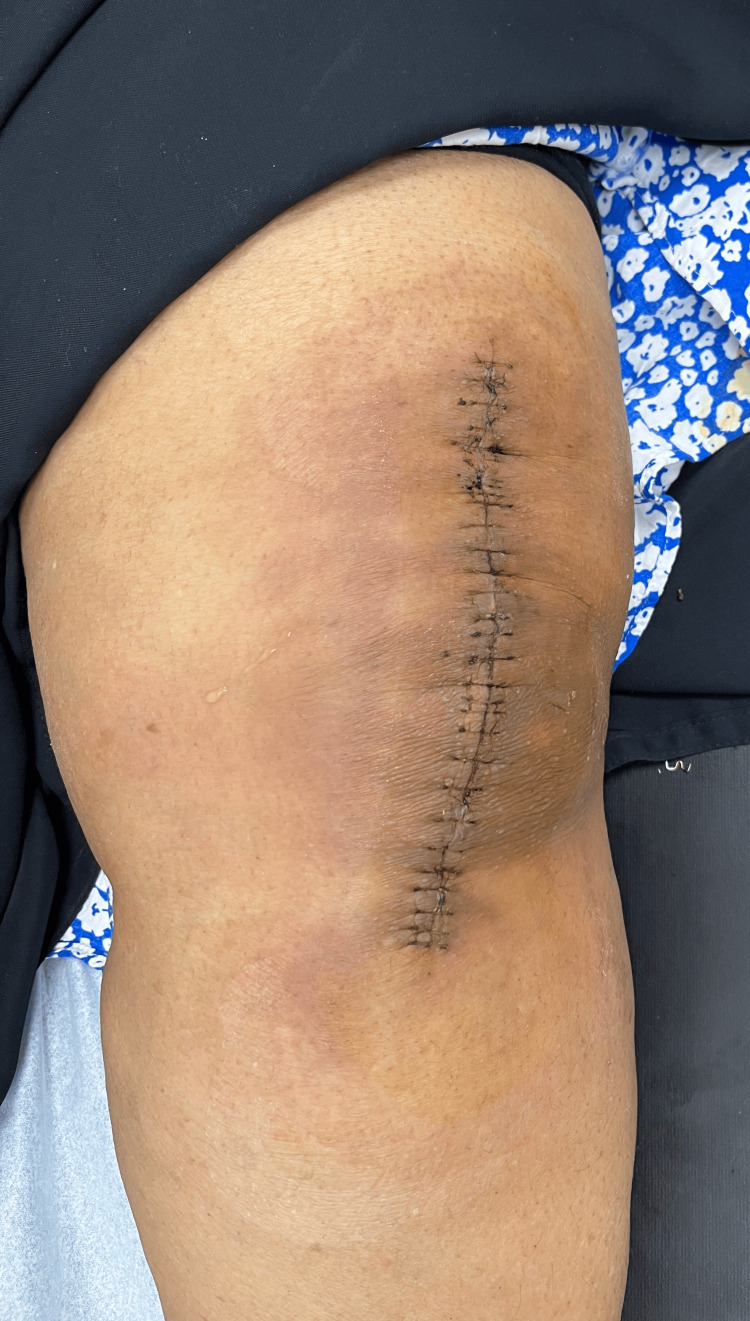
Wound at three weeks postoperatively, with staples removed

**Figure 6:**
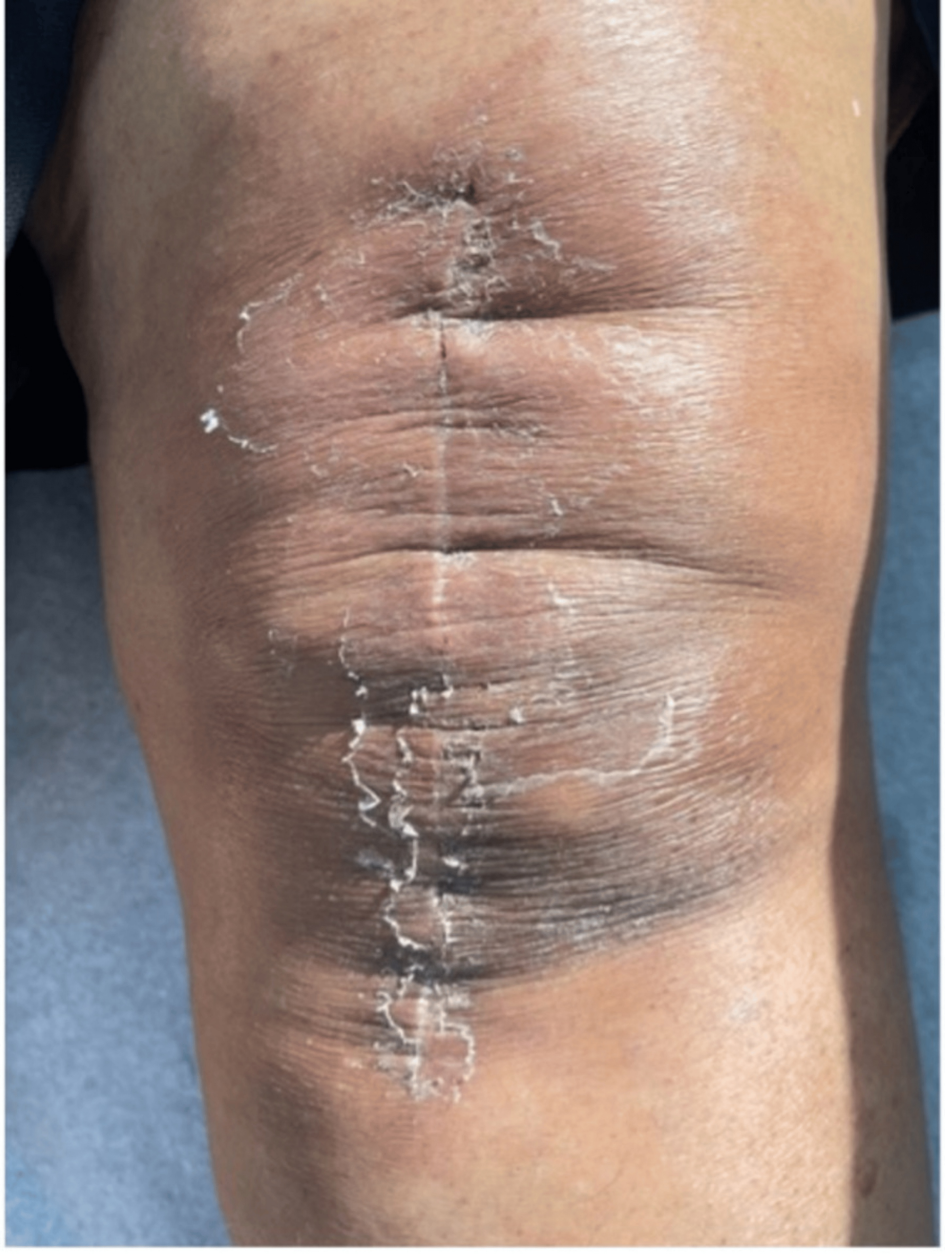
Wound at four weeks postoperatively, with the surgical scar healing

**Figure 7:**
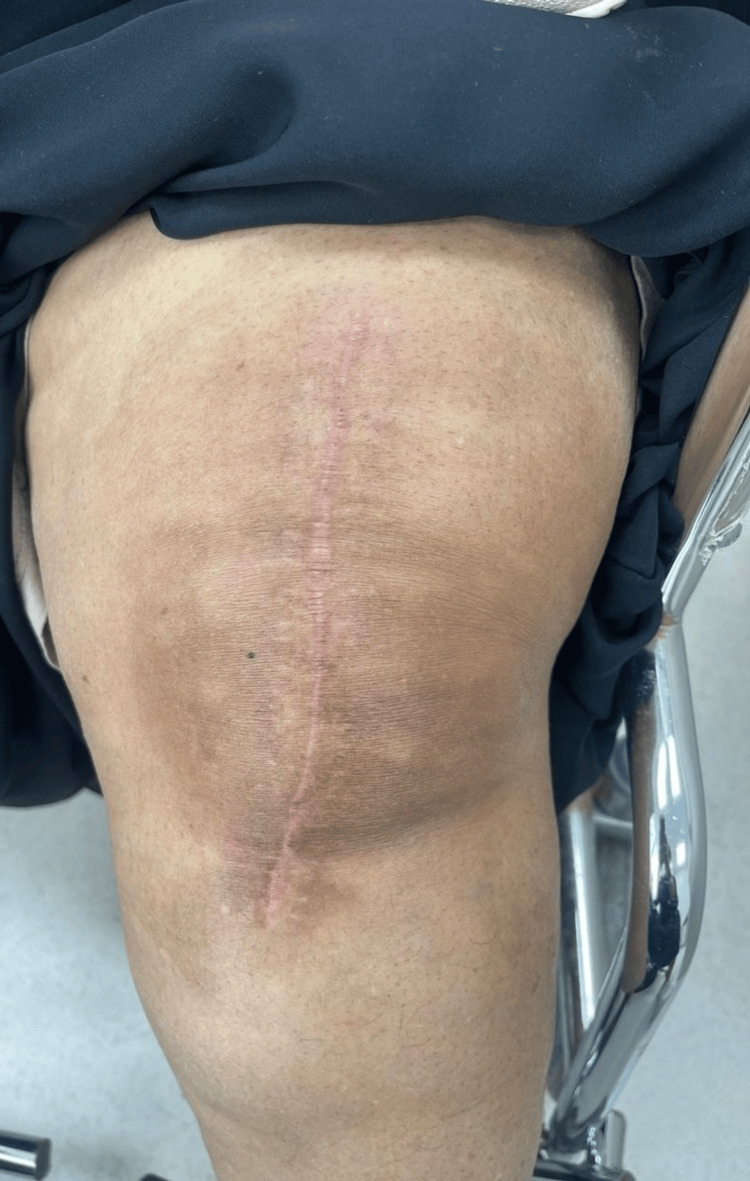
Wound at 12 weeks postoperatively, with the surgical scar healing

The lesions resolved after changing the silver sulfate-containing wound dressing; it was replaced with one not containing silver sulfate. Moreover, she was prescribed loratadine (Claritin) 10 mg one tablet a day for two weeks, dimetindene (Fenistil) topical ointment twice a day for two weeks, and cephalexin (Keflex) 500 mg two tablets a day as prophylaxis. The patient denied previous contact with silver or history of silver allergy. Moreover, no prior use of this dressing or contact with silver-containing agents was known to this patient. Patch testing was positive for silver allergic contact dermatitis. The patient came to the clinic for five follow-up visits at 1 week (Figure 3), 2 weeks (Figure 4), 3 weeks (Figure 5), 4 weeks (Figure 6), and 12 weeks (Figure 7). Throughout this period, the lesions resolved after changing the silver sulfate-containing wound dressing (Figure 4). Furthermore, the staples were removed on her third follow-up visit (Figure 5). To the best of our knowledge, this is the only case reported on ACD from silver sulfate present in a wound dressing.

## Discussion

Silver-containing wound dressings are widely utilized in postoperative care due to their anti-bacterial, anti-inflammatory, and anti-oxidative properties, which aid in wound healing and prevention of surgical site infections [5]. However, their use rarely leads to adverse reactions, such as allergic contact dermatitis. ACD is a delayed-type hypersensitivity reaction mediated by T cells in response to contact allergens, including metals like silver [6].

In this case, the patient developed erythema, itching, and burning sensations localized to the area where the Mepilex Border Post-Op Ag dressing, containing silver sulfate, was applied. The resolution of symptoms upon removal of the dressing and positive patch testing suggests a localized allergic response rather than an irritant reaction or infection [7].

The pathophysiology of ACD involves the penetration of silver ions into the epidermis, where they bind to skin proteins, forming haptens that activate antigen-presenting cells and trigger a T-cell-mediated immune response. This mechanism aligns with the delayed onset of ACD symptoms, typically occurring days after exposure [8].

While silver-induced ACD is relatively rare, clinicians should be aware of this potential complication, especially in patients presenting with localized inflammatory reactions following the application of silver-containing dressings. Early recognition and prompt removal of the offending agent are crucial to prevent further complications and to facilitate appropriate management.

## Conclusions

This case highlights the need to consider allergic contact dermatitis as a potential adverse reaction to silver sulfate-containing wound dressings. Healthcare providers should remain vigilant for ACD in patients who develop localized dermatitis following the use of silver-based dressings, particularly those with known or suspected metal hypersensitivities. In this specific case, the patient was diabetic and at increased risk of secondary bacterial infections, further emphasizing the importance of selecting appropriate wound care options.
